# Thermal stability data of silver nanowire transparent conducting electrode

**DOI:** 10.1016/j.dib.2020.105422

**Published:** 2020-03-12

**Authors:** Choong-Heui Chung, Taejun Park, Sangyeob Lee

**Affiliations:** Department of Materials Science and Engineering, Hanbat National University, Daejeon 34158, Republic of Korea

**Keywords:** Silver nanowire, Transparent conducting electrode, Electrodeposition, Rayleigh instability, CIGS solar cell

## Abstract

The authors have recently reported the enhanced thermal stability of silver nanowire (AgNW) network transparent electrodes by electrodeposition method [1]. AgNW networks are known to break into droplets at elevated temperatures (spherodization temperature) that are still much lower than the bulk Ag melting temperature. This phenomenon is known as Rayleigh instability. As the diameter of individual AgNW in the network increases by electrodeposited Ag on the AgNW surface, the thermal stability of AgNW network can be enhanced. Here, we provide the data on the spherodization temperature depending on AgNW diameter. We also report the calculated activation energy required to induce the spherodization of AgNW network.

Specifications table

**Table:** 

Subject	Materials Science
*Specific subject area*	Transparent conducting electrodes
*Type of data*	Scanning electron microscope (SEM) images and Figures
*How data were acquired*	SEM image (FE-SEM, HITACHI SU5000) Temperature and resistance (Keithley 2700 multimeter data acquisition system with sample under N_2_ environment)
*Data format*	Raw and analyzed
*Parameters for data collection*	The resistance by increasing temperature The AgNW diameter controlled by electrodeposition time
*Description of data collection*	The resistances of AgNW network were measured in N_2_ environment with increasing temperature. The spherodization temperatures were determined by the temperature at which the AgNW network displayed no conductance. The diameter of AgNW network was enlarged by increasing the AgNW electrodeposition time.
*Data source location*	Hanbat National University, Daejeon 34158, Republic of Korea
*Data accessibility*	Raw data related to Fig. 2(a) and (b) are available within this article as a supplementary file. SEM images are included in the article.
*Related research article*	Author's name: Sangyeob Lee et al. Title: Electrodeposited silver nanowire transparent conducting electrodes for thin-film solar cells Journal: ACS Applied Materials & Interfaces DOI: 10.1021/acsami.9b17168

## Value of the data

• To utilize a AgNW network as a reliable component in a optoelectrical device, the thermal stability is critical issue to be resolved. The stability of AgNW network can be enhanced by increasing the diameter of AgNW. Therefore, it is required to have data regarding the effect of diameter on the thermal stability of AgNW network.
• Detailed information of critical diameters and temperatures maintaining the reliability of AgNW network can provide a knowledge to understand the failure mechanism of AgNW network.
• The AgNW network is a potential candidate for next generation transparent conducting electrodes (TCEs) of solar cells, specifically Cu(In,Ga)Se_2_ thin film solar cell. The data can be useful for device/process engineers who are responsible for TCE process for a solar cell to determine the TCE postprocess temperature.

## 1 Data description

**Fig. 1:**
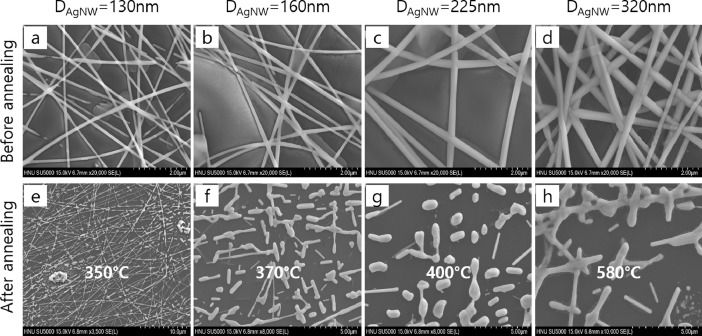
SEM images of AgNW networks with AgNW diameters of (a) 130 nm, (b) 160 nm, (c) 225 nm, and (d) 320 nm before annealing. SEM images of AgNW network of (a)–(d) after annealing at (e) 350 °C, (f) 370 °C, (g) 400 °C, and (h) 580 °C. AgNW diameters were increased from bare AgNW network with diameter of 80 nm by Ag electrodeposition.

**Fig. 2:**
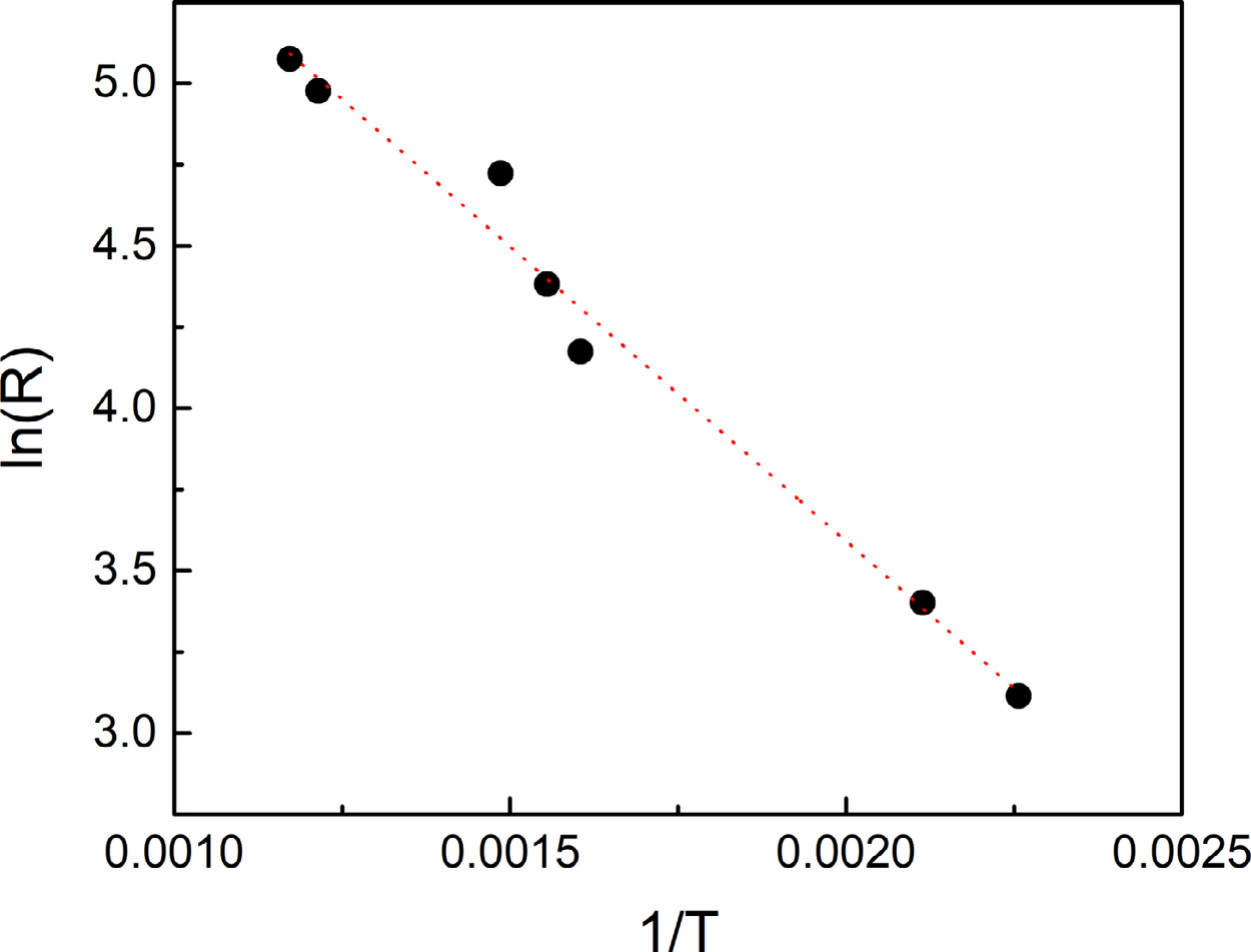
The failure radius of ED AgNW vs. spherodization temperature plot for the calculation of the activation energy for surface diffusion.

The diameter of AgNWs in a network can be increased by Ag electrodeposition on a spin-coated bare AgNW network. The diameter of AgNWs can be precisely controlled by adjusting electrodeposition time [1]. The electrodeposited (ED) AgNW networks with different diameters are shown in the scanning electron microscope (SEM) images of Fig. 1(a)–(d). Fig. 1(e)–(h) show SEM images of the ED AgNW network after annealing in a N_2_ atmosphere. The AgNW networks are broken into droplets at elevated temperatures. We defined the temperature at which the network is broken into droplets and starts to show no conductance as the spherodization temperature. As the diameter of AgNW increases, the spherodization temperature also increases showing the improved thermal stability. We measured the spherodization temperature of the ED AgNW network as a function of the diameter of the AgNWs and verified the failure mechanism of AgNWs network by plotting the radius and spherodization temperature as shown in Fig. 2. The failure time of a AgNW network due to atomic surface diffusion can be defined using the following equation

(1) $$\tau_{m}=\left(4R^{4}kT/\gamma_{S}D_{0}\right)exp\left(-\frac{Q}{kT}\right)$$

(2) $$4\ln R=\;ln\left(\frac{\tau_{m}\gamma_{S}D_{0}}{kT}\right)-\;\left(\frac{Q}{kT}\right)$$

where *τ_m_* is the characteristic time (failure time), *R* is the radius of the NW, *k* is the Boltzmann constant, *T* is the temperature, *γ_s_* is the surface energy, *D_0_* is the pre-factor and *Q* is the activation energy [2,3]. The activation energy of surface diffusion can be calculated by Eq. (2), which is derived from Eq. (1). Using Eq. (2), the activation energy for surface diffusion in AgNWs is obtained, and its value is 0.15 eV, which is similar to the value for silver adatom diffusion on a Ag surface [4], indicating that AgNW failure occurs due to Ag atomic diffusion through the AgNW surface.

## 2 Experimental design, materials, and methods

### 2.1 ED AgNW TCE fabrication

A AgNW TCE is fabricated by two steps. First, a bare AgNW TCE is fabricated by spin-coating of AgNWs solution onto a 25 mm × 25 mm glass at 1000 rpm for 30 s. The initial diameter of AgNWs is 80 nm before electrodeposition. The AgNW solution is prepared by diluting AgNWs in ethanol at a concentration of 20 mg/ml. After preparing the spin-coated AgNW on glass, the diameter of AgNWs is increased by electrodeposition of Ag onto AgNW surfaces. For electrodeposition, the silver electrolyte is prepared by mixing of 70 g/L of silver cyanide, 150 g/L of potassium cyanide, 15 g/L of potassium carbonate, and 50 g/L of sodium thiosulfate. AgNW spin-coated glass and Ag foil are used as the working and counter electrodes, respectively. A constant current density of 1 mA/cm^2^ is applied for several deposition cycles using a potentiostat. The diameter of AgNWs can be increased by repeating the Ag electrodeposition cycles.

### 2.2 Measurement of spherodization temperature

The AgNW diameter of ED AgNW network is measured using a field emission SEM. The spherodization temperatures of the ED AgNW network with various diameters are measured using a Keithley 2700 multimeter data acquisition system with samples under N_2_ environment. The temperature and resistance of the ED AgNW network are monitored simultaneously as increasing the temperature. The spherodization temperature is recorded when the ED AgNW network shows no conductance.

## Conflict of Interest

The authors declare that they have no kwon competing financial interests of personal relationships that could have appeared to influence the work reported in this paper.

## Appendix Supplementary material

Image, application 1

## References

[1] Lee S., Jang J., Park T., Park Y.M., Park J.S., Kim Y.-K., Lee H.-K., Jeon E.-C., Lee D.-K., Ahn B., Chung C.-H. (2020). Electrodeposited silver nanowire transparent conducting electrodes for thin-film solar cells. ACS Appl. Mater. Interfaces.

[2] Nichols F.A. (1976). On the spherodization of rod-shaped particles of finite length. J. Mater. Sci..

[3] Jiran E., Thompson C.V. (1990). Capillary instabilities in thin films. JEM.

[4] Papanicolaou N.I., Evangelakis G.A., Kallinteris G.C. (1998). Molecular dynamic description of silver adatom diffusioin on Ag(100) and Ag(111) surfaces. Comput. Mater. Sci..

