# Supplementary material for: Thermal stability data of silver nanowire transparent conducting electrode
Source: Data Brief. 2020 Mar 12;30:105422. doi: 10.1016/j.dib.2020.105422 (PMC7109625; doi:10.1016/j.dib.2020.105422)
Supplement: Supplementary file 1 [file mmc1.pdf]

X [1/T]<br>T = Spherodization temperature	Y [ln(R)]<br>R = Radius of AgNW
0.00226	3.11352
0.00211	3.4012
0.00161	4.17439
0.00156	4.38203
0.00149	4.72295
0.00122	4.97673
0.00117	5.07517
